# Validation of Osteoporosis associated SNPs candidates from Decisive Gene Strategy using Taiwan Biobank

**DOI:** 10.7150/ijms.125797

**Published:** 2026-08-24

**Authors:** Pei-Jan Tsai, Sin-Yu Lin, Meng-Chang Lee, Mei-Jung Chen, Chih-Chien Wang, Cheng-Jung Chen, Wei-Teing Chen, Po-Jen Hsiao, Chih-Chien Chiu, Yu-Hsuan Chen, Hsiao-Ting Lin, Hao Su, Sui-Lung Su

**Affiliations:** 1 Graduate Institute of Medical Sciences, College of Medicine, National Defense Medical University, Taipei 11490, Taiwan, R.O.C.; 2 Graduate Institute of Aerospace and Undersea Medicine, College of Biomedical Sciences, National Defense Medical University, Taipei 114201, Taiwan, R.O.C.; 3 Graduate Institute of Public Health, College of Public Health, National Defense Medical University, Taipei 114201, Taiwan, R.O.C.; 4 Department of Nursing, Tri-Service General Hospital, National Defense Medical University, Taipei 114202, Taiwan, R.O.C.; 5 School of Medicine, College of Medicine, National Defense Medical University, Taipei, Taiwan, R.O.C.; 6 Department of Orthopedics, Tri-Service General Hospital, National Defense Medical University, Taipei 114202, Taiwan, R.O.C.; 7 Division of General Surgery, Department of Surgery, Tri-Service General Hospital, National Defense Medical University, Taipei 114202, Taiwan, R.O.C.; 8 Department of Surgery, Chiayi Branch, Taichung Veterans General Hospital, Chiayi City 60090, Taiwan, R.O.C.; 9 Division of Thoracic Medicine, Department of Medicine, Cheng Hsin General Hospital, Taipei, Taiwan, R.O.C.; 10 Division of Nephrology, Department of Internal Medicine, Taoyuan Armed Forces General Hospital, Taoyuan, Taiwan, R.O.C.; 11 Division of Nephrology, Department of Internal Medicine, Tri-Service General Hospital, National Defense Medical University, Taipei, Taiwan, R.O.C.; 12 Division of Infectious Diseases, Department of Internal Medicine, Taoyuan Armed Forces General Hospital, Taoyuan, Taiwan, R.O.C.; 13 Graduate Institute of Life Sciences, College of Biomedical Sciences, National Defense Medical University, Taipei 114201, Taiwan, R.O.C.

**Keywords:** osteoporosis, single nucleotide polymorphisms, decisive gene strategy, Taiwan Biobank

## Abstract

Decisive gene strategy (DGS) has been developed and identified 21 single nucleotide polymorphisms (SNPs) associated with osteoporosis (OP). This study aimed to further evaluate whether DGS is a suitable candidate strategy to identify the relationship between SNPs and OP using Taiwan Biobank (TWB). A total of 26,225 samples were retrieved from TWB between 2008 and 2021, comprising 4,749 osteoporosis (T-score ≤ -2.5) and 21,476 controls (T-score ≥ -1.0) based on their T-score of bone mineral density (BMD). Logistic regression analysis was used to examine the association between 21 SNPs from DGS and OP risk by using TWB data. The alignment of 21 SNPs from DGS with TWB genotyping chip found 11 identical SNPs. Among the 11 identical SNPs, seven showed results consistent with prior DGS evidence, including ESR1 rs9340799, OPG rs2073617, VDR rs731236, rs7975232, and rs1544410, IL6 rs1800795, and IGF1 rs5742612 [OR: 1.01, 1.03, 1.06, 1.01, 1.10, 0.72, 1.02; 95% CI= 0.94-1.09, 0.97-1.10, 0.93-1.22, 0.94-1.07, 0.97-1.26, 0.32-1.64, 0.96-1.09, respectively]. Furthermore, OPG T245G rs3134069 showed a borderline nominal association with osteoporosis in TWB [OR: 1.09, 95% CI= 1.00-1.19]), suggesting a potential population-specific signal. Our TWB validation of the 11 identical SNPs supports DGS as an effective methodology and, beyond replication, provides a novel framework for examining the relationship between SNPs and disease.

## 1. Introduction

Osteoporosis (OP) is a major public health issue characterized by low bone mineral density (BMD) and structural deterioration 1, affects 20% global population 2, 3 and ranks as the fourth most common chronic disease among Taiwanese elderly 4, 5. Significant evidence showed BMD variation have high heritability 6 and associated with single nucleotide polymorphisms (SNPs) in several genes 7, 8.

Meta-analysis ranks the highest in the evidence hierarchy in medical research studies and provides evidence for clinical practice and healthcare. Numerous SNPs such as estrogen receptor α (ESR1) XbaI rs9340799, ESR1 PvuII rs2234693, estrogen receptor β (ESR2) RsaI rs1256049, ESR2 AluI rs4986938, osteoprotegerin (OPG) T950C rs2073617, OPG G1181C rs2073618, OPG A163G rs3102735, vitamin D receptor (VDR) ApaI rs7975232, BsmI rs1544410, TaqI rs731236, and FokI rs10735810 polymorphisms, have been identified significantly associated with osteoporosis through meta-analyses 9-14. However, previous meta-analyses on gene polymorphisms and diseases may include too few studies and patients to obtain sufficient statistical power, leading to spurious positive results 15 and increase Type I errors 16. Trial sequential analysis (TSA) has been developed to resolve these problems. TSA addresses this concern by providing a mechanism to halt sample accumulation in conventional meta-analyses, and by using visual aids to determine whether further sampling is necessary 17-18.

Our previous study has comprehensively screened meta-analysis articles on OP and identified 21 candidate gene loci in Caucasian and Asian ethnic population. Additionally, we conduct TSA to examine the sufficiency of the cumulative sample size for each gene locus to determine whether a definitive conclusion could be drawn to OP. This novel methodology which combines "keyword search" and "trial sequential analysis (TSA)" approaches are named as Decisive Gene Strategy (DGS), which may overcome meta-analysis limitation and offer novel therapeutic targets 19. More evidence is needed to support whether DGS is a robust strategy to seek the relationship between SNPs and OP. We hypothesized that DGS-prioritized SNPs would be validated in the Taiwan Biobank, with effect directions consistent with prior DGS-derived evidence. Therefore, this study aimed to further evaluate whether DGS is a suitable candidate strategy to the relationship between SNPs and osteoporosis using Taiwan Biobank (TWB).

## 2. Materials and Methods

### 2.1 Study participants

Taiwan Biobank (TWB) is a population-based research consortium. The study population is comprised of 153,443 TWB Han Chinese participants who had no history of cancer and were recruited between 2008 and 2021 from centers across Taiwan. We excluded a total of 127,218 participants due to (1) SNPs data non-available (37,377 participants), (2) BMD data non-available (77,049 participants), and (3) Osteopenia (-2.5 < T-score < -1.0) samples (12,792 participants). There were 26,225 individuals enrolled to our study, and participants were divided to case (T-score ≤ -2.5) and control (T-score ≥ -1.0) group. This study was approved by the Institutional Review Board of the Tri-Service General Hospital (TSGH), a medical teaching hospital of the National Defense Medical Centre in Taipei, Taiwan (approval number: C202305088, approval date: 1 August 2023), was conducted in compliance with the ethical standards of national and institutional committees on human research and the Helsinki Declaration.

### 2.2 Bone mineral density (BMD) measurements

Bone mineral density (BMD) was assessed at the calcaneus (heel bone) using an ultrasound device (Achilles InSight, GE) and expressed in grams per square centimetres (g/cm²). Osteoporosis was defined by a T-score of less than -2.5 and classified as case group, while individuals with T-scores greater than -1.0 were normal bone density and classified in control group. It is well established that T-scores at or below -2.5 are indicative of osteoporosis, signifying substantially lower bone density and an elevated risk of fractures and related complications 20. In contrast, T-scores above -1.0 are considered within the normal range, reflecting healthy bone density. T-scores between -2.5 and -1.0 represent osteopenia, a condition characterized by lower bone density but not severe enough to meet the criteria for osteoporosis. Due to the distinct clinical management required for osteopenia and osteoporosis, individuals with osteopenia were excluded from our study.

### 2.3 Alignment of SNPs from DGS with TWB genotyping chip

#### TWB genotyping chip

TWB integrates individual genotype data with comprehensive clinical information. Genomic DNA was extracted from peripheral blood, and genotyping was performed within the Taiwan Biobank framework. The customized TWB1 genotyping array is based on the Thermo Fisher Axiom Genome-Wide CHB array, enabling the detection of approximately 650,000 variants related to cancer risk and common polymorphisms in Taiwanese population. Subsequently, leveraging genotyping data from TWB1 array and whole-genome sequencing (WGS) data from approximately 1,000 TWB participants, the NCGM and Thermo Fisher Scientific collaboratively developed the TWB2 genotyping array, specifically tailored for Taiwanese population. The TWB2 array includes ~750,000 variants, with ~100,000 shared with TWB1, and is enriched with rare coding variants, making it a valuable resource for clinical and precision medicine research. Genotype processing, including phasing, imputation using a combined 1000 Genomes East Asian and TWB whole-genome sequencing reference panel, and standard quality control procedures (e.g., imputation quality, allele frequency, call rate, relatedness, and principal component analysis-based ancestry screening), followed the established TWB pipeline as previously described 21. Previous TWB studies have also shown that the cohort is composed predominantly of Han Chinese individuals and is genetically relatively homogeneous 21-23.

#### Decisive Gene Strategy (DGS)

The Decisive Gene Strategy (DGS) methodology integrates keyword search and trial sequential analysis (TSA) to evaluate the association between gene polymorphisms and osteoporosis 19. Initially, a systematic keyword search of SCI-indexed publications was conducted to identify meta-analyses on gene polymorphisms related to osteoporosis. Gene distribution frequencies from various loci were then extracted from the identified studies. TSA was subsequently applied to assess the adequacy of cumulative sample sizes, determining whether specific gene loci acted as protective factors, risk factors, or showed no association with osteoporosis. Through this dual approach, DGS provides a comprehensive overview of gene loci implicated in OP.

#### SNPs grouping after alignment with TWB chip

Previous study has identified 21 SNPs associated with OP through DGS 19. These 21 SNPs are aligned to TWB genotyping chip (TWB1+TWB2) by using R software, and the alignment results are classified as three groups: (1) **Identical SNPs group**: which found the same rs number SNPs, (2) **High linkage disequilibrium (LD) SNPs group**: which found SNPs show r^2^ ≥ 0.8, and (3)** Non-available SNPs group**: which SNPs were not found in genotyping chip.

### 2.4 Statistical analysis

Categorical data were evaluated using the chi-square test. Student's t test was used to compare the difference in the means from two continuous variables. Logistic regression analysis was performed to infer the association of SNPs with OP, with adjustment for age, sex, and BMI; principal components were not included as covariates in the regression models since principal component analysis had already been incorporated into the TWB pipeline. The criterion for significance was set at p value < 0.05 for all tests. All statistical analyses were performed using R software, version 4.2.1. Genetic association analyses were conducted using custom R scripts, including SNP alignment, genetic model coding, and logistic regression analyses.

## 3. Results

### 3.1 Subject characteristics

A total of 26,225 samples (4,749 OP cases and 21,476 controls) were recruited to this study, flow chart of inclusion is shown in **Figure 1**. Significant differences were observed between cases and control groups in sex, age, BMI, and T-score variables. Female were dominant in both groups, in which 2,975 (62.6%) of cases and 14,225 (66.2%) of controls. Mean age was significantly higher in the case group (61.25 ± 8.40 years old) than control group (52.09 ± 10.31 years old). Mean BMI was significantly lower in cases (24.63 ± 3.81 kg/m^2^) than in controls (23.70 ± 3.66 kg/m^2^). Additionally, mean T-score was significantly lower in cases (-3.04 ± 0.51) compared to controls (0.41 ± 1.17), results shown in **Table 1**.

### 3.2 SNPs from DGS alignment with TWB chip

Alignment of the 21 SNPs from DGS with TWB genotyping chip found 11 identical SNPs in TWB chip, including rs9340799, rs1800795, rs2073617, rs3134069, rs731236, rs7975232, rs1544410, rs2288377, rs5742612, rs4986938, and rs1256049. Additionally, 6 SNPs exhibited high LD (r² ≥ 0.8): rs2234693, rs3102735, rs2073618, rs3736228, rs35767, and rs1800470. However, 4 SNPs (rs2228480, rs2228570, rs1107946, and rs1800012) were non-available in TWB chip. The alignment flowchart is presented in **Figure 2**. Genotype distributions and case-control counts for the 11 identical and 6 high-LD SNPs are provided in **Supplementary Table 1**, and no marked differences were observed between cases and controls.

### 3.3 Logistic regression analysis of SNPs from DGS

**Table 2** shows the logistic regression analysis of the 11 identical SNPs associated with OP in TWB samples. Among them, rs3134069 showed a nominal risk association to OP in both the allele model [OR=1.09, 95% CI=1.00-1.19, p=0.05] and dominant model [OR=1.10, 95% CI=1.00-1.21, p=0.05]. However, the remaining 10 SNPs showed no significant association in the allele, dominant, or recessive models in overall samples.

**Supplementary Table 2** provides the index-proxy mapping (including r²) for the 6 high-LD SNPs and the complete logistic regression results across genetic models in TWB. rs3102735 showed a nominal protective association with osteoporosis in dominant model [OR=0.81, 95% CI=0.69-0.95, p=0.01], while rs3736228 showed a nominal protective association in recessive model [OR=0.81, 95% CI=0.69-0.95, p=0.01]. The remaining 4 SNPs showed no nominal significant association across allele, dominant, or recessive models. The 4 non-available SNPs were not analyzed.

### 3.4 Logistic regression analysis of SNPs from DGS stratified by gender and age

Further gender- and age-stratified analyses were conducted to investigate the association between the 11 identical SNPs and OP. In overall male samples, no nominal association was observed under the allele or dominant model, whereas rs1544410 showed a nominal association with OP under recessive model [OR=3.02, 95% CI=1.28-7.10, p=0.01]. No nominal association were observed in male under 65 years old. In male aged over 65 years old, rs9340799 showed a nominal protective association with OP in both allele [OR=0.78, 95% CI=0.63-0.96, p=0.02] and dominant model [0.78, 95% CI=0.60-0.96, p=0.02], while rs4986938 showed a nominal risk association in both allele [OR=1.34, 95% CI=1.02-1.75, p=0.03] and dominant model [OR=1.38, 95% CI=1.03-1.84, p=0.03]. Additionally, rs1256049 demonstrated a borderline nominal protective association in the allele model [OR=0.85, 95% CI=0.72-1.00, p=0.05]. The complete male sex- and age-stratified regression outputs for the 11 identical SNPs are provided in **Supplementary Table 3**.

In overall female samples, no nominal association was observed in allele and dominant model, while rs1256049 showed a nominal protective association with OP in recessive model [OR=0.85, 95% CI=0.74-0.97, p=0.02]. In female under 65 years old, rs1256049 showed nominal protective association in allele [OR=0.91, 95% CI=0.84-0.99, p=0.02] and recessive model [OR=0.84, 95% CI=0.71-0.99, p=0.04]. In female aged over 65 years old, rs2288377 was nominally associated with OP under the allele [OR= 1.18, 95% CI= 1.06-1.33, p < 0.01], dominant [OR= 1.23, 95% CI= 1.06-1.44, p < 0.01), and recessive model [OR= 1.27, 95% CI= 1.00-1.61, p<0.01]. Similarly, rs5742612 showed nominal association under the allele [OR=1.18, 95% CI=1.06-1.33, p<0.01], dominant [OR=1.23, 95% CI=1.06-1.44, p<0.01], and recessive models [OR=1.27, 95% CI=1.00-1.61, p<0.01]. Conversely, rs9340799 showed nominal protective association in both allele [OR=0.84, 95% CI=0.73-0.96, p<0.01] and dominant model [OR=0.81, 95% CI=0.69-0.95, p=0.01], and rs7975232 showed a nominal protective association in dominant model [OR=0.85, 95% CI=0.73-0.99, p=0.04]. The corresponding female sex- and age-stratified regression outputs for the 11 identical SNPs are detailed in **Supplementary Table 4**.

Gender- and age-stratified analyses of the 6 high LD SNPs are shown below. Male sex- and age-stratified results across genetic models are provided in **Supplementary Table 5**. In overall sample, rs3736228 showed a nominal association in both allele [OR=1.11, 95% CI=1.00-1.22, p=0.04] and recessive model [OR=1.40, 95% CI=1.05-1.85, p=0.02], and rs35767 showed a nominal association in dominant model [OR=1.13, 95% CI=1.01-1.26, p=0.03]. In male under 65 years old, rs3736228 also showed a nominal association in recessive model [OR=1.41, 95% CI=1.03-1.93, p=0.03], while other SNPs showed no significant association. In male aged over 65 years old, rs1800470 showed nominal protective association in recessive model [OR=0.73, 95% CI=0.54-0.99, p=0.04]. Female sex- and age-stratified results across genetic models are provided in **Supplementary Table 6**. In the overall sample, no SNP was significant associated with OP in both overall and under 65 years old sample. In female aged over 65 years old, rs35767 showed a nominal association in both allele [OR=1.14, 95% CI=1.02-1.28, p=0.02] and dominant model [OR=1.21, 95% CI=1.03-1.41, p=0.02].

### 3.5 Validation results of SNPs from DGS using TWB samples

**Table 3** shows the validation result in TWB of the 11 identical and 6 high LD SNPs which previously aligned with TWB genotyping chip. Allele frequencies of these aligned variants in TWB were generally similar to those reported for the 1000 Genomes Project East Asian (EAS) populations (**Supplementary Table 7**), supporting the population relevance of our findings in an East Asian context. For the 11 identical SNPs group, 7 SNPs of which showed consistent result with previous DGS findings in the overall sample, which are ESR1 XbaI rs9340799, OPG T950C rs2073617, VDR TaqI rs731236, VDR ApaI rs7975232, VDR BsmI rs1544410, IL6 G174C rs1800795, and IGF1 rs5742612. Our replication analysis showed that OPG T245G rs3134069 had a nominal risk association with OP [OR=1.09, 95% CI=1.00-1.19, p=0.05], suggests potential new genetic contributors to osteoporosis susceptibility. However, 3 SNPs, ESR2 RsaI rs1256049, ESR2 AluI rs4986938 and IGF1 rs2288377, showed inconsistent validation result with previous DGS finding.

For the 6 high LD SNPs, 3 SNPs of which showed consistent result with previous DGS findings in the overall samples (ESR1 PvuII rs2234693, OPG G1181C rs2073618), and male samples (LRP5 rs3736228). However, our replication results to the other 3 SNPs, rs3102735, rs35767 and rs1800470, showed inconsistent validation result with previous DGS finding.

## 4. Discussion

The alignment of SNPs from DGS with TWB genotyping chip found 11 identical and 6 high LD SNPs. Overall, TWB validation showed both concordant findings and discordant results relative to DGS conclusions. Among the 11 identical SNPs, 7 SNPs demonstrated consistent results with previous DGS evidence in TWB, whereas a subset showed inconsistency. Notably, 1 SNP was identified as novel association in our study (OPG T245G rs3134069), highlighting potential new genetic contributors to osteoporosis risk. Allele-frequency differences for some variants may partly contribute to variability in transferability across populations and should be considered when interpreting cross-ethnic comparisons. For the 6 high LD SNPs, 3 SNPs demonstrated consistent results with previous DGS studies in the overall or sex-stratified analyses, whereas the remaining variants did not.

### Seven identical SNPs exhibit consistent results with previous DGS findings

#### ESR1 (rs9340799)

ESR1 encodes estrogen receptor-α and is expressed in osteoblasts, osteoclasts, and bone marrow stromal cells, supporting its biological plausibility for osteoporosis through estrogen-mediated regulation of bone remodeling 24. Previous DGS study both included 7 research of ESR1 XbaI rs9340799 in Caucasians and Asians with 1,839 and 3,123 accumulated samples respectively. Results showed that cumulative sample size were sufficient to confirm ESR1 XbaI rs9340799 are not associated with OP in Caucasians and Asians. Our validation in TWB showed consistent result with previous DGS study that ESR1 XbaI rs9340799 polymorphism did not exhibit a significant association with OP in overall samples. In age-stratified analysis, significant protective effects were observed in both males and females aged over 65 years, suggesting that the effect of rs9340799 may be age dependent. One possible explanation is that this variant becomes more relevant in the hormonal milieu of older individuals, particularly under relatively low-estrogen conditions. Since ESR1/ERα is a major mediator of estrogen action in bone 25, and estrogen deficiency has been associated with altered bone turnover and RANKL/OPG-related regulation 26, rs9340799 may modulate ESR1 sensitivity or downstream signaling under low-estrogen conditions, thereby influencing the RANKL/OPG balance and bone resorption. Although estrogen may be one possible explanation for the protective effect observed in women aged over 65 years, the similar association found in men of the same age group suggests that additional mechanisms may be involved and warrant further investigation. These findings suggest that rs9340799 is unlikely to be a strong determinant of osteoporosis risk in the overall cohort, although it may still exert age-specific protective effects in elderly individuals.

#### OPG (rs2073617)

OPG is a secreted glycoprotein, and its main mechanism is to inhibit osteoclast differentiation and maturation 27-28. Previous DGS study found 1 research of OPG T950C rs2073617 with 555 samples in Caucasians, while no related research in Asians was found. Results showed that OPG T950C rs2073617 are not significant associated with OP in Caucasians, while accumulate sample size are needed to draw confirm conclusion. Our validation in TWB showed consistent result with previous DGS study that OPG T950C rs2073617 polymorphism did not exhibit a significant association with OP in overall samples. These findings are consistent with the DGS-inferred null association for rs2073617, suggesting that any effect of this common OPG variant, despite the biological relevance of the OPG/RANKL/RANK pathway in osteoclast regulation, may be modest in TWB.

#### VDR (rs731236, rs7975232, and rs1544410)

VDR polymorphisms are biologically plausible in osteoporosis because vitamin D-VDR signaling regulates calcium and phosphate homeostasis, intestinal calcium absorption, and the transcription of osteogenesis-related genes, thereby influencing bone mineralization and remodeling 29-30. For VDR TaqI rs731236, previous DGS study found 5 research of VDR TaqI rs731236 with 1,056 accumulated samples in Caucasians, while no related research in Asians was found. Results showed that VDR TaqI rs731236 are not significant associated with OP in Caucasians, while accumulate sample size are needed to draw confirm conclusion. Our validation in TWB showed consistent result with previous DGS study that VDR TaqI rs731236 polymorphism did not exhibit a significant association with OP in overall samples. These findings suggest that the effect of rs731236 may be modest and population dependent, and additional validation in Asian cohorts would be helpful; however, because the prior evidence was derived from Caucasian cohorts, transferability to TWB should be interpreted cautiously given potential population differences.

For VDR ApaI rs7975232, previous DGS study both found 7 research of VDR ApaI rs7975232 in Caucasians and Asians with 1,728 and 1,804 accumulated samples respectively. Results showed that VDR ApaI rs7975232 are not associated with OP in Caucasians and Asians, while only Caucasians reached sufficient cumulate sample size. Our validation in TWB showed consistent result with previous DGS study that VDR ApaI rs7975232 polymorphism did not exhibit a significant association with OP in overall samples. These results suggest that effect of rs7975232 is likely modest and may vary across populations; therefore, additional population-specific studies with adequate cumulative sample sizes would be helpful to clarify its contribution to osteoporosis risk.

For VDR BsmI rs1544410, previous DGS study found 16 (3,620 samples) and 19 (2,473 samples) research of VDR BsmI rs1544410 in Caucasians and Asians respectively. Results showed that VDR BsmI rs1544410 are not associated with OP in Caucasians and Asians, while only Caucasians reached sufficient cumulate sample size. Our validation in TWB showed consistent result with previous DGS study that VDR BsmI rs1544410 polymorphism did not exhibit a significant association with OP in overall samples. Our findings suggest that rs1544410 polymorphisms does not show a robust association with osteoporosis in TWB, any genetic effects may be modest and sensitive to population-specific factors. However, stratified analysis revealed a significant association in males under the recessive model (OR = 3.02, p = 0.01), suggesting that the overall null result may obscure a sex-dependent genetic effect. One possible explanation is that regulatory variation in the VDR locus may affect vitamin D signaling and downstream pathways involved in bone remodeling 31-32. This effect may be more evident in males, in whom testosterone and muscle mass are important determinants of bone maintenance; thus, disruption of the vitamin D/VDR axis may have a greater impact on bone loss in men 33-34. Further functional and sex-stratified studies are needed to clarify the mechanism underlying this association.

#### IL6 (rs1800795)

Interleukin-6 (IL-6) gene encodes a multifunctional cytokine that plays a critical role in stimulating both the formation and resorption of bone cells 35-36. Previous DGS study found 9 (7,536 samples) and 1 (318 samples) research of IL-6 G174C rs1800795 in Caucasians and Asians respectively. Results showed that cumulative sample size were sufficient to confirm IL-6 G174C rs1800795 are not associated with OP in Caucasians and Asians. Our validation in TWB showed consistent result with previous DGS study that IL-6 G174C rs1800795 polymorphism did not exhibit a significant association with OP in overall samples.

#### IGF1 (rs5742612)

Insulin-like growth factor-1 (IGF1) is the primary ligand for IGF receptor 1 (IGF1R) 37, which acts as the key mediator of growth hormone (GH) effects on bone growth and mineralization 38. Previous DGS study found 6 research of IGF1 rs5742612 with 4,031 samples in Asians, while no related research in Caucasians was found. Results showed that cumulative sample size were sufficient to confirm IGF1 rs5742612 are not associated with OP in Asians. Our validation in TWB showed consistent result with previous DGS study that IGF1 rs5742612 polymorphism did not exhibit a significant association with OP in overall samples.

### Three identical SNPs exhibit inconsistent results with previous DGS findings

#### ESR2 (rs1256049 and rs4986938)

ESR2 also plays a vital role in bone metabolism 24. Previous DGS study both found 1 research of ESR2 RsaI rs1256049 in Caucasians and Asians with 380 and 1,190 accumulated samples respectively. Results showed that cumulative sample size were sufficient to confirm ESR2 RsaI rs1256049 are not associated with OP in Caucasians, while a protective SNP to Asians. However, in our validation study, no significant association was observed, which is inconsistent with previous DGS findings. A possible explanation for this discrepancy with prior reports is that the DGS study included only female participants 39, whereas the TWB cohort comprised both males and females. Accordingly, we cannot exclude the possibility that differences in sex composition may have contributed to the observed inconsistency between studies.

Previous DGS study found 3 (1,097 samples) and 1 (1,303 samples) research of ESR2 AluI rs4986938 in Caucasians and Asians respectively. Results showed that ESR2 AluI rs4986938 are significant associated with OP in Asians but not in Caucasians, while accumulate sample size are needed to draw confirm conclusion. However, our validation study did not show a significant association, which is inconsistent with the DGS findings. A possible explanation for this discrepancy with prior reports is that the TSA did not yield conclusive evidence in the prior DGS study, and that the DGS sample included only female participants 39, whereas the TWB cohort comprised both males and females. Thus, we cannot exclude the possibility that limited conclusiveness from TSA and differences in sex composition may have contributed to the observed inconsistency between studies.

#### IGF1 (rs2288377)

Previous DGS study found 6 research of IGF1 rs2288377 with 4,029 samples in Asians, while no related research in Caucasians was found. Results showed that cumulative sample size were sufficient to confirm IGF1 rs2288377 polymorphism is associated with OP in Asians. However, our validation study showed no significant association, inconsistent with the DGS findings. A possible explanation is that the DGS evidence was largely derived from cohorts of postmenopausal women with an average age over 65 years, whereas the TWB participants were relatively younger (cases: 61.25 ± 8.40 years; controls: 52.09 ± 10.31 years). Accordingly, we cannot exclude the possibility that differences in age distribution and menopausal status may have reduced our ability to detect an association and contributed to the non-significant overall result in TWB.

### One identical SNP reveals novel finding to OP in Taiwanese

#### OPG (rs3134069)

OPG encodes osteoprotegerin, a secreted decoy receptor that binds RANKL and inhibits RANKL-RANK signaling, thereby suppressing osteoclast differentiation and bone resorption, a central pathway in bone remodeling 40. Previous DGS study found 2 research of OPG T245G rs3134069 with 596 samples in Caucasians, while no related research in Asians was found. Results showed that cumulative sample size were sufficient to confirm OPG T245G rs3134069 are not associated with OP in Caucasians. In our validation using TWB data, rs3134069 showed a borderline nominal association in overall samples. This may represent a potential population-specific signal in Taiwanese population; however, the modest effect size and borderline p value warrant cautious interpretation. One possible explanation is that the promoter variant rs3134069 in TNFRSF11B/OPG may influence OPG expression 41. Reduced OPG expression could weaken its inhibitory effect on RANKL, thereby enhancing RANKL/RANK signaling and downstream NF-κB and MAPK activation, which promote osteoclast differentiation and bone resorption 42-43. In addition, because transcription factor-related mechanisms, including Sp1- and AP-1-related regulations, have been implicated in OPG promoter regulation 44, this variant may contribute to osteoporosis risk through altered transcriptional regulation of OPG. Nevertheless, whether rs3134069 directly affects Sp1 or AP-1 binding has not been conclusively determined and requires further functional validation 45. Given the modest effect size in TWB, this finding should be interpreted cautiously and warrants independent replication in additional Asian cohorts. Further functional studies examining whether rs3134069 is associated with OPG expression or circulating OPG levels would help clarify the underlying mechanism.

### Three high-LD SNPs exhibit consistent results with previous DGS findings

#### ESR1 (rs2234693)

Previous DGS study both found 7 research of ESR1 PvuII rs2234693 in Caucasians and Asians with 1,726 and 3,010 accumulated samples respectively. Results showed that cumulative sample size were sufficient to confirm ESR1 PvuII rs2234693 polymorphism is associated with OP in Caucasians. No association was found in Asians, while accumulate sample size are needed to draw confirm conclusion. Our validation in TWB showed consistent result with previous DGS study that ESR1 PvuII rs2234693 polymorphism did not exhibit a significant association with OP in overall samples. Accordingly, the null result in TWB does not exclude the biological relevance of ESR1 but suggests that any effect of this common variant may be modest and population dependent.

#### OPG (rs2073618)

Previous DGS study found 1 research of OPG G1181C rs2073618 with 555 samples in Caucasians, while no related research in Asians was found. Results showed that OPG G1181C rs2073618 are not significant associated with OP in Caucasians, while accumulate sample size are needed to draw confirm conclusion. Our validation in TWB showed consistent result with previous DGS study that OPG G1181C rs2073618 polymorphism did not exhibit a significant association with OP in overall samples. These findings suggest that while the OPG pathway is biologically relevant to bone remodeling, rs2073618 may not represent a robust determinant of osteoporosis risk in this cohort. Because the prior evidence base was derived mainly from non-Asian cohorts and our TWB analysis relied on an LD proxy.

#### LRP5 (rs3736228)

Lipoprotein receptor-related protein5 (LRP5) is a single-pass transmembrane receptor expressed in bone and is biologically relevant to osteoporosis through Wnt-mediated regulation of osteoblast function and bone formation 46-47. Previous DGS study found 2 research of LRP5 A1330V rs3736228 with 481 samples in Caucasians, while no related research in Asians was found. Results showed that LRP5 A1330V rs3736228 are significant associated with OP in Caucasians, while accumulate sample size are needed to draw confirm conclusion. We further evaluated these two studies and found that 164 out of 481 participants (34.1%) were male 48-49, which is similar to the male proportion in our TWB analytic sample (9,025/26,225; 34.4%) and may provide a plausible context for the observed consistency in male-stratified analysis.

### Three high-LD SNPs exhibit inconsistent results with previous DGS findings

#### OPG (rs3102735)

Previous DGS study found 2 research of OPG A163G rs3102735 with 624 samples in Caucasians, while no related research in Asians was found. Results showed that OPG A163G rs3102735 are significant associated with OP in Caucasians, while accumulate sample size are needed to draw confirm conclusion. However, our validation study did not identify a significant association, which is inconsistent with previous DGS results. The observed discrepancy may be partly attributable to differences between our TWB cohort and the Caucasian populations included in the DGS literature. Given that the DGS findings were based on only two studies with a relatively small sample size (n = 624) and that the TSA did not provide conclusive evidence, additional studies in Asian populations would be helpful to clarify this association. These interpretations should be considered cautiously given potential population differences.

#### IGF1 (rs35767)

Previous DGS study found 7 research of IGF1 rs35767 with 4,575 samples in Asians, while no related research in Caucasians was found. Results showed that cumulative sample size were sufficient to confirm IGF1 rs35767 polymorphism is associated with OP in Asians. However, our validation study did not demonstrate a significant association, differing from the results obtained through DGS. One potential factor is that the proportion of female participants was higher in the seven studies (81.64%) than in our TWB sample (66%). Thus, differences in sex composition may have contributed to the observed inconsistency between studies.

#### TGFβ1 (rs1800470)

Transforming growth factor-β1 (TGFβ1) is a bone-derived factor 50, which is associated with osteoclast growth, thereby affecting bone resorption and recovery 51-52. Previous DGS study found 3 (972 samples) and 7 (3,472 samples) research of TGFβ1 T869C rs1800470 in Caucasians and Asians respectively. Results showed that cumulative sample size were sufficient to confirm TGFβ1 T869C rs1800470 polymorphism is associated with OP in Asians. No association was found in Caucasians, while accumulate sample size are needed to draw confirm conclusion. However, our validation study did not demonstrate a significant association, which was not consistent with the association reported in prior Asian studies summarized by the DGS; notably, our TWB analysis relied on a high-LD proxy (rs2241715) rather than the original rs1800470. Accordingly, we cannot exclude the possibility that rs2241715 is an imperfect proxy for rs1800470 in this cohort.

Our study evaluated 17 SNPs across several genetic models and subgroup analyses; no multiple-testing correction were conducted due to two considerations. First, the 17 SNPs evaluated in this study were not identified through an exploratory genome-wide screening approach; rather, they were prespecified candidate variants rigorously prioritized through the DGS, which incorporates extensive meta-analyses and TSA. In the context of an independent external validation cohort TWB, the primary objective was to evaluate whether the association of these candidate variants with osteoporosis could be reproduced. Second, applying highly stringent multiple-testing correction in this targeted validation context may be overly conservative and substantially increase the risk of type II error, potentially obscuring modest but genuine genetic effects that are characteristic of complex traits such as osteoporosis. Therefore, we considered nominal significance (p < 0.05), together with consistency in effect direction and biological plausibility, to be a more appropriate framework for interpretation in this validation study.

### Strengths and Limitations

Our study has four strengths. First, this study developed an innovative method termed Decisive Gene Strategy (DGS). Unlike traditional meta-analysis approaches that typically focus on individual SNPs, the DGS method integrates information from multiple disease-related genes and loci, enabling a more comprehensive evaluation of genetic associations. Second, by incorporating TSA, the proposed approach accounts for cumulative sample size and statistical reliability, allowing for more robust and conclusive assessments of SNP-disease associations. This strategy enhances the internal validity of the findings and reduces the likelihood of spurious results. Third, the performance of the DGS approach was further validated using external data from the TWB. Several SNPs prioritized by DGS were successfully replicated in this independent cohort, supporting the feasibility, reproducibility, and generalizability of the method in real-world populations. Finally, DGS facilitates the prioritization of disease-related SNPs and may help reduce false-positive findings in genome-wide association studies (GWAS). The prioritized variants can be further incorporated into machine learning models to estimate their relative contributions, with predictive performance evaluated using the area under the receiver operating characteristic curve (AUC), sensitivity, and specificity. This framework supports the development of personalized risk prediction models for osteoporosis.

Our study still has some limitations. First, this was a hypothesis-driven replication of DGS-prioritized SNPs rather than an exploratory genome-wide discovery analysis; moreover, the replication component still has limited statistical power. Second, the average age of TWB samples is relatively young, which may underestimate the impact on specific SNPs and osteoporosis. Third, our cohort consists exclusively of Han Chinese participants; thus, comparisons with predominantly Caucasian evidence in the DGS literature should be interpreted cautiously due to potential ancestry differences (e.g., allele frequencies and LD structure), which may partly contribute to non-replication. Fourth, osteoporosis status was defined using calcaneal quantitative ultrasound (QUS) to derive T-scores rather than dual-energy X-ray absorptiometry (DXA), which may be less precise and introduce outcome misclassification (QUS AUC = 0.737, using DXA as the reference standard) that could attenuate observed associations. Future studies with central DXA measurements would be helpful for further validation.

## 5. Conclusion

Our validation of the 11 identical and 6 high LD SNPs from DGS using TWB demonstrates that DGS is an effective methodology. While confirming the DGS findings, our validation also offers a novel framework for examining the relationship between SNPs and disease.

## Supplementary Material

Supplementary tables.

## Figures and Tables

**Figure 1:**
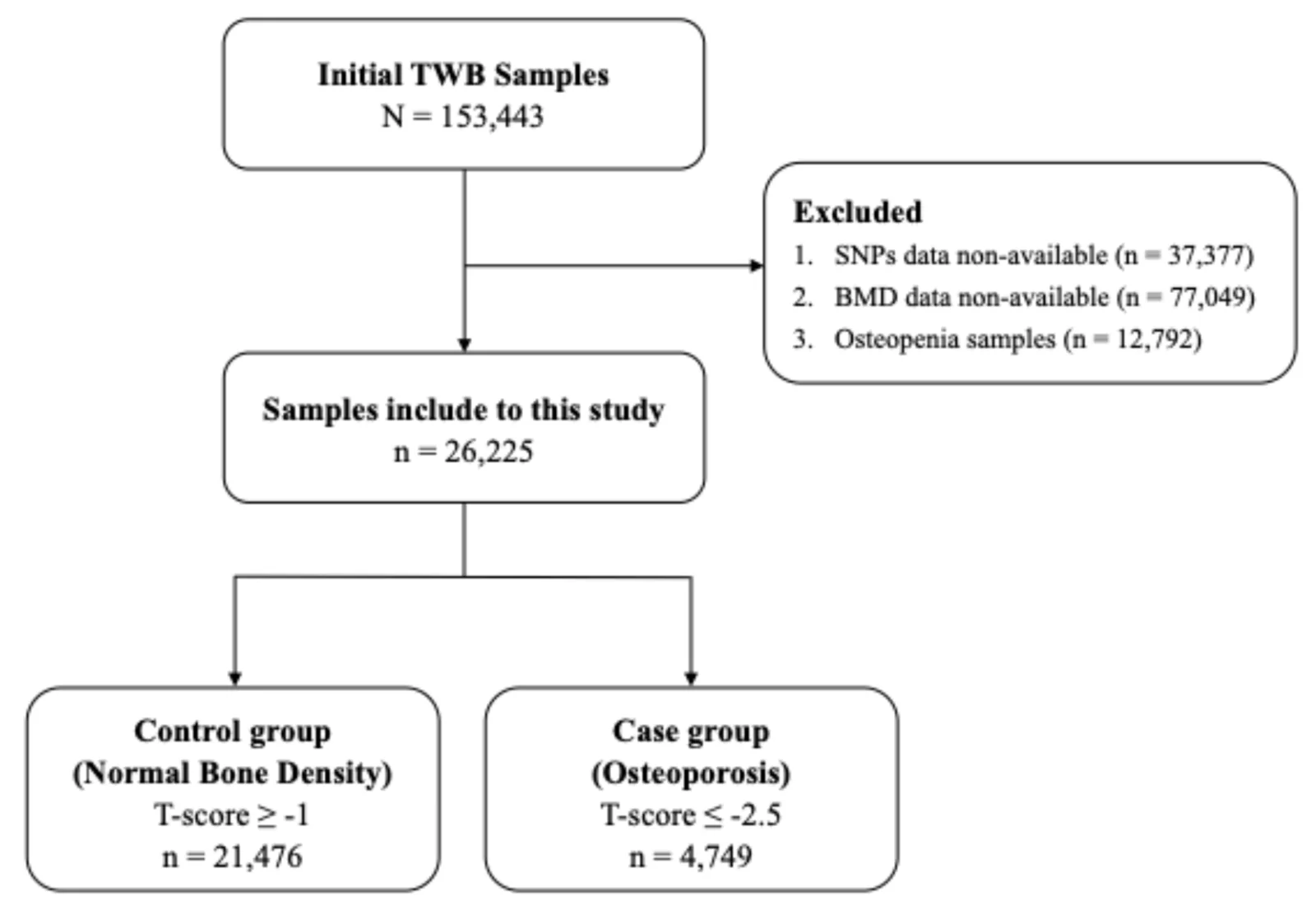
** Flow chart of the participants included in the TWB.** This study recruited 153,443 individuals from TWB. After excluded 37,377 individuals which SNPs data are non-available, 77,049 individuals BMD data are non-available (using calcaneus ultrasound device for BMD measurement), and 12,792 individuals classified as osteopenia (-1 < T-score < -2.5), a total of 26,225 individuals were included in this study. These individuals were then divided according to T-score. 21,476 normal BMD individuals were classified as control group, while 4,749 osteoporosis individuals were classified as case group. Abbreviation: TWB, Taiwan Biobank; SNPs, single nucleotide polymorphisms; BMD, bone mineral density.

**Figure 2:**
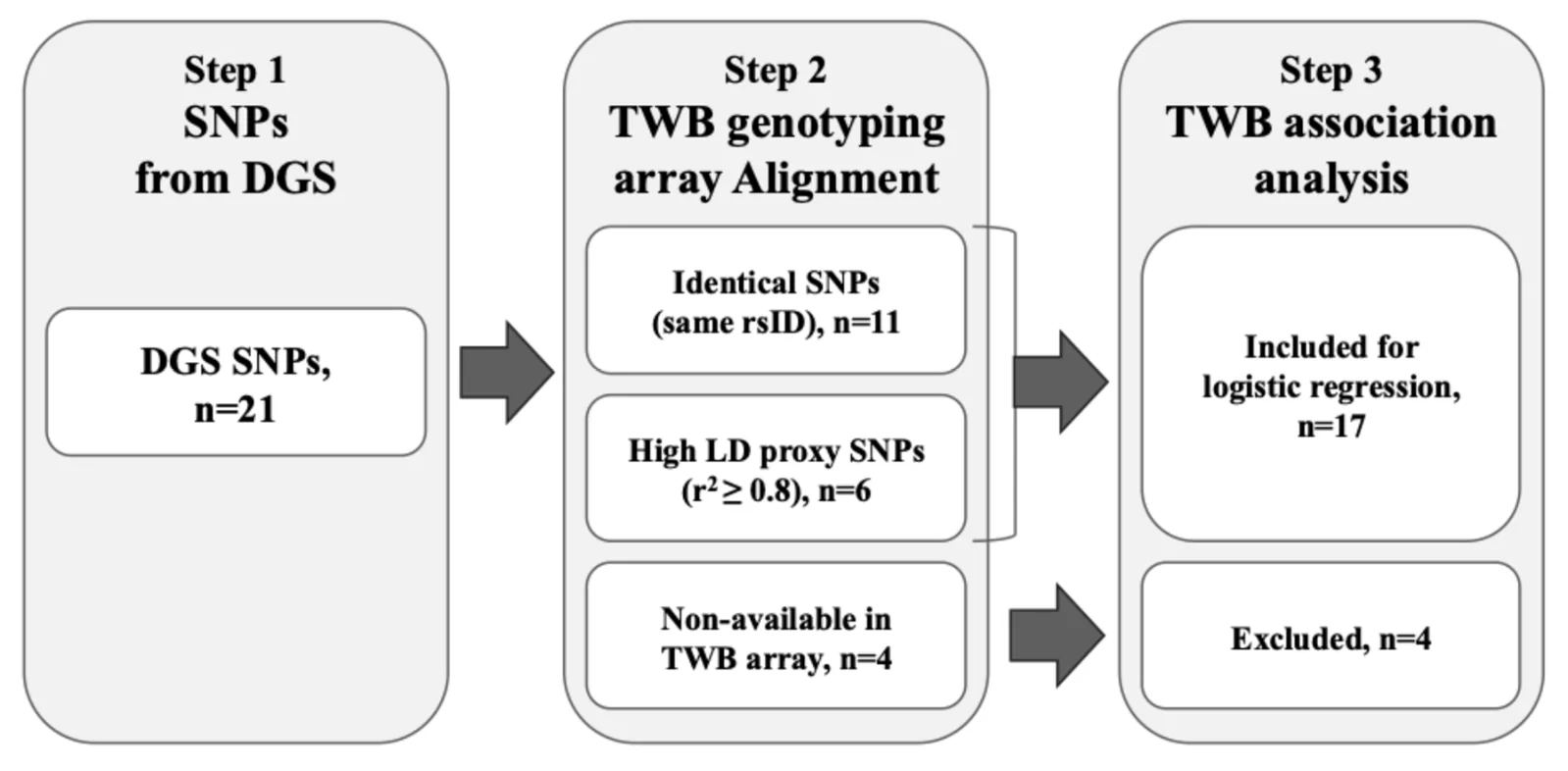
** Flowchart of aligning DGS-identified SNPs with the TWB genotyping array.** Twenty-one osteoporosis-associated SNPs were identified by the Decisive Gene Strategy (DGS) 19 and mapped to the Taiwan Biobank (TWB) genotyping array. Among these 21 SNPs, 11 were directly available with identical rsIDs (identical SNPs), 6 were represented by proxy variants in high linkage disequilibrium (LD; r² ≥ 0.8), and 4 were not available on the TWB array. The 17 available SNPs (identical and high-LD proxies) were forwarded to logistic regression in the TWB case-control study. Abbreviations: DGS, Decisive Gene Strategy; OP, osteoporosis; SNP, single-nucleotide polymorphism; TWB, Taiwan Biobank; LD, linkage disequilibrium.

**Table 1:** Demographics of the study population

Variables	Control group (n=21,476)	Case group (n=4,749)	p-value
Gender			<0.001*
Male	7,251 (33.8%)	1,774 (37.4%)	
Female	14,225 (66.2%)	2,975 (62.6%)	
Age (years old)	52.09 ± 10.31	61.25 ± 8.40	<0.001*
BMI (kg/m^2^)	24.63 ± 3.81	23.70 ± 3.66	<0.001*
T-score	0.41 ± 1.17	-3.04 ± 0.51	<0.001*

Data are shown as mean ± SD or number (%). Abbreviation: TWB, Taiwan Biobank; BMI, body mass index. *p<0.05

**Table 2:** Logistic regression analysis of 11 identical SNPs in TWB to justify their relationship to OP

SNPs	CHR	Major/Minor	Allele		Dominant		Recessive	
			OR (95% CI)	p-value	OR (95% CI)	p-value	OR (95% CI)	p-value
rs9340799	6	A/G	1.01 (0.94-1.09)	0.72	1.01 (0.93-1.10)	0.83	1.05 (0.86-1.28)	0.63
rs1800795	7	C/G	0.72 (0.32-1.64)	0.44	0.68 (0.30-1.54)	0.35	NA	NA
rs2073617	8	G/A	1.03 (0.97-1.10)	0.33	1.04 (0.95-1.13)	0.43	1.05 (0.93-1.18)	0.43
rs3134069	8	A/C	1.09 (1.00-1.19)	0.05*	1.10 (1.00-1.21)	0.05*	1.11 (0.82-1.50)	0.49
rs731236	12	A/G	1.06 (0.93-1.22)	0.40	1.07 (0.93-1.24)	0.34	0.83 (0.34-2.04)	0.68
rs7975232	12	C/A	1.01 (0.94-1.07)	0.85	1.00 (0.92-1.09)	0.96	1.03 (0.88-1.20)	0.72
rs1544410	12	C/T	1.10 (0.97-1.26)	0.14	1.10 (0.96-1.26)	0.16	1.32 (0.60-2.89)	0.50
rs2288377	12	A/T	1.02 (0.96-1.09)	0.58	1.01 (0.93-1.10)	0.84	1.07 (0.93-1.23)	0.36
rs5742612	12	A/G	1.02 (0.96-1.09)	0.58	1.01 (0.93-1.10)	0.84	1.07 (0.93-1.23)	0.36
rs4986938	14	C/T	1.02 (0.92-1.13)	0.71	1.03 (0.93-1.15)	0.56	0.86 (0.56-1.33)	0.50
rs1256049	14	C/T	0.95 (0.90-1.01)	0.12	0.95 (0.88-1.04)	0.27	0.91 (0.81-1.03)	0.14

*p<0.05, adjusted with age, gender, and BMI. Allele, Dominant, and Recessive genetic models were evaluated. Allele: per-minor-allele effect (0/1/2); Dominant: (Aa + aa) vs AA; Recessive: aa vs (AA + Aa). Abbreviations: DGS, decisive gene strategy; SNPs, single-nucleotide polymorphisms; TWB, Taiwan Biobank; CHR, chromosome

**Table 3:** Validation of SNPs from DGS to the risk of OP using TWB samples

SNPs from DGS	rs number	TSA in Caucasians		TSA in Asians		TWB samples validation^c^			Validation result^e^	TWB chip alignment
		OR (95%CI)	I^2^	OR (95%CI)	I^2^	Overall OR (95%CI)	Male OR^d^ (95%CI)	Female OR^d^ (95%CI)		
*ESR1* *XbaI*	rs9340799	0.87^a^ (0.58-1.31)	88%	0.86^a^ (0.39-1.89)	97%	1.01 (0.94-1.09)	0.94 (0.85-1.03)	0.95 (0.88-1.02)	Consistent with overall	Identical
*OPG* *T950C*	rs2073617	0.93^b^ (0.73-1.17)	0%	No meta research		1.03 (0.97-1.10)	1.04 (0.96-1.13)	1.01 (0.95-1.08)	Consistent with overall	Identical
*VDR* *TaqI*	rs731236	1.34^b^ (0.94-1.92)	68%	No meta research		1.06 (0.93-1.22)	1.06 (0.88-1.26)	0.95 (0.82-1.11)	Consistent with overall	Identical
*VDR* *ApaI*	rs7975232	0.90^a^ (0.72-1.36)	72%	1.21^b^ (0.81-1.80)	81%	1.01 (0.94-1.07)	1.02 (0.94-1.11)	0.96 (0.90-1.03)	Consistent with overall	Identical
*VDR* *BsmI*	rs1544410	0.92^a^ (0.76-1.11)	64%	1.01^b^ (0.64-1.60)	85%	1.10 (0.97-1.26)	1.15 (0.98-1.36)	0.96 (0.83-1.11)	Consistent with overall	Identical
*IL6* *G174C*	rs1800795	0.94^a^ (0.87-1.01)	0%	0.61^a^ (0.05-7.28)	0%	0.72 (0.32-1.64)	0.60 (0.13-2.82)	0.88 (0.32-2.41)	Consistent with overall	Identical
*IGF1*	rs5742612	No meta research		1.10^a^ (0.97-1.26)	0%	1.02 (0.96-1.09)	1.03 (0.95-1.12)	1.04 (0.97-1.12)	Consistent with overall	Identical
*ESR1* *PvuII*	rs2234693	1.06^a^ (0.75-1.50)	82%	0.82^b^ (0.55-1.22)	90%	1.01 (0.95-1.07)	1.00 (0.92-1.08)	0.98 (0.91-1.04)	Consistent with overall	High LD, rs3853250
*OPG* *G1181C*	rs2073618	0.87^b^ (0.70-1.10)	0%	No meta research		0.98 (0.92-1.05)	1.00 (0.91-1.09)	0.98 (0.91-1.05)	Consistent with overall	High LD, rs7464496
*LRP5* *A1330V*	rs3736228	1.50^b^ (1.08-2.07)	0%	No meta research		1.06 (0.98-1.14)	1.11* (1.00-1.22)	1.05 (0.97-1.14)	Consistent in male	High LD, rs3781586
*ESR2* *RsaI*	rs1256049	1.30^a^ (0.81-2.10)	0%	0.69^a^ (0.59-0.81)	0%	0.95 (0.90-1.01)	0.96 (0.88-1.04)	0.94 (0.88-1.00)	Inconsistent	Identical
*ESR2* *AluI*	rs4986938	1.23^b^ (0.58-2.57)	94%	1.31^b^ (1.05-1.64)	0%	1.02 (0.92-1.13)	1.05 (0.92-1.19)	1.03 (0.92-1.15)	Inconsistent	Identical
*IGF1*	rs2288377	No meta research		1.44^a^ (1.28-1.62)	0%	1.02 (0.96-1.09)	1.03 (0.95-1.12)	1.04 (0.97-1.12)	Inconsistent	Identical
*OPG* *A163G*	rs3102735	1.49^b^ (1.11-2.00)	0%	No meta research		1.09 (1.00-1.18)	1.06 (0.96-1.18)	1.08 (0.99-1.19)	Inconsistent	High LD, rs1385504
*IGF1*	rs35767	No meta research		1.20^a^ (1.06-1.36)	47%	1.03 (0.96-1.09)	1.07 (0.99-1.16)	1.02 (0.96-1.09)	Inconsistent	High LD, rs1101820
*TGFβ1* *T869C*	rs1800470	1.03^b^ (0.58-1.83)	70%	1.35^a^ (1.10-1.65)	74%	0.98 (0.92-1.04)	0.99 (0.92-1.07)	0.98 (0.92-1.05)	Inconsistent	High LD, rs2241715
*OPG* *T245G*	rs3134069	0.79^a^ (0.06-10.28)	68%	No meta research		1.09* (1.00-1.19)	1.07 (0.96-1.20)	1.08 (0.98-1.18)	Novel finding	Identical

^a^ Sufficient cumulative sample size to confirm association between SNPs and OP. ^b^ Still need to accumulate samples to confirm association between SNPs and OP. ^c^ The OR of TWB samples validation demonstrate allele model of overall cohort and sex-stratified analyses (male and female). Allele: per-minor-allele effect (0/1/2). ^d^ Gender and age stratified analysis through Taiwan Biobank case-control study samples.^e^ Validation results were categorized as Consistent (consistent with the DGS conclusion, including replicated associations with concordant direction and statistical significance in TWB as well as consistent null findings), Inconsistent (directionally inconsistent and/or inconsistent with the DGS conclusion), or Novel finding (statistically significant in TWB but not supported by the DGS conclusion). ^*^ p<0.05 Abbreviations: TSA, trial sequential analysis; ESR1, estrogen receptor 1; ESR2, estrogen receptor 2; OPG, osteoprotegerin; VDR, vitamin D receptor; IGF-1, insulin-like growth factor-1; IL6, interleukin-6; LRP5, lipoprotein receptor-related protein5; TGFβ1, transforming growth factor-β1.

## Data Availability

Data are available from the authors upon reasonable request and with permission of Taiwan Biobank. To facilitate reproducibility, summary statistics (ORs and SEs of ln [OR]) for the overall and sex-stratified analyses are provided in Supplementary Table 8. Analysis scripts may be made available from the corresponding author upon reasonable request, subject to institutional and Taiwan Biobank data-use regulations.
