# Supplementary material for: Validation of Osteoporosis associated SNPs candidates from Decisive Gene Strategy using Taiwan Biobank
Source: Int J Med Sci. 2026 Aug 24;23(10):3122–33. doi: 10.7150/ijms.125797 (PMC13617501; doi:10.7150/ijms.125797)
Supplement: Supplementary file 1 [file ijmsv23p3122s1.pdf]

Supplementary Table 1. Genotype distribution of 11 identical and 6 high LD SNPs in TWB case-control samples

SNPs	Aligned result	Control group (n=21,476)	Case group (n=4,749)	p-value
rs9340799_A/G	Identical			0.216
AA		13263(61.8%)	2995(63.1%)	
AG		7259(33.8%)	1543(32.5%)	
GG		954(4.4%)	211(4.4%)	
rs1800795_C/G	Identical			0.962
CC		21434(99.8%)	4739(99.8%)	
CG		40(0.2%)	9(0.2%)	
GG		-	-	
rs2073617_G/A	Identical			0.764
GG		8509(39.8%)	1865(39.5%)	
GA		9988(46.8%)	2204(46.7%)	
AA		2863(13.4%)	651(13.8%)	
rs3134069_A/C	Identical			0.092
AA		16257(75.7%)	3529(74.4%)	
AC		4836(22.5%)	1120(23.6%)	
CC		376(1.8%)	97(2.0%)	
rs731236_A/G	Identical			0.563
AA		19437(90.6%)	4316(90.9%)	
AG		1960(9.1%)	421(8.9%)	
GG		56(0.3%)	9(0.2%)	
rs7975232_C/A	Identical			0.42
CC		10822(50.6%)	2428(51.3%)	
CA		8862(41.4%)	1913(40.4%)	
AA		1715(8.0%)	393(8.3%)	
rs1544410_C/T	Identical			0.373
CC		19274(90.0%)	4266(89.9%)	
CT		2098(9.8%)	463(9.8%)	
TT		52(0.2%)	17(0.4%)	
rs2288377_A/T	Identical			0.54
AA		10288(47.9%)	2234(47.1%)	
AT		9087(42.3%)	2035(42.9%)	
TT		2097(9.8%)	478(10.1%)	
rs5742612_A/G	Identical			0.533
AA		10289(47.9%)	2234(47.0%)	
AG		9089(42.3%)	2037(42.9%)	
GG		2098(9.8%)	478(10.1%)	
rs4986938_C/T	Identical			0.86
CC		17798(82.9%)	3920(82.5%)	
CT		3481(16.2%)	785(16.5%)	
TT		197(0.9%)	44(0.9%)	
rs1256049_C/T	Identical			0.362
CC		8560(39.9%)	1917(40.4%)	
CT		9945(46.3%)	2212(46.6%)	
TT		2971(13.8%)	620(13.1%)	
rs3853250_T/G	High LD			0.892
TT		7951(37.5%)	1762(37.5%)	
TG		10047(47.4%)	2235(47.6%)	
GG		3219(15.2%)	700(14.9%)	
rs1385504_T/A	High LD			0.074
TT		15973(74.4%)	3456(72.8%)	
TA		5064(23.6%)	1182(24.9%)	
AA		429(2.0%)	106(2.2%)	
rs7464496_T/C	High LD			0.628
TT		12194(56.8%)	2735(57.6%)	
TC		8011(37.3%)	1742(36.7%)	
CC		1249(5.8%)	271(5.7%)	
rs3781586_C/A	High LD			0.105
CC		14260(66.5%)	3094(65.2%)	

CA		6470(30.2%)	1468(31.0%)	
AA		708(3.3%)	180(3.8%)	
rs1101820_C/T	High LD			0.116
CC		9077(42.8%)	1939(41.3%)	
CT		9506(44.8%)	2181(46.4%)	
TT		2628(12.4%)	579(12.3%)	
rs2241715_A/C	High LD			0.87
AA		6706(31.2%)	1477(31.1%)	
AC		10521(49.0%)	2345(49.4%)	
CC		4249(19.8%)	927(19.5%)	

SNPs are listed in order of chromosome position.
Data are shown as number (%).
Abbreviations: DGS, decisive gene strategy; SNPs, single-nucleotide polymorphisms; TWB, Taiwan Biobank; LD, linkage disequilibrium
*p<0.05

Supplementary Table 2. Logistic regression analysis of 6 high LD SNPs to justify their relationship to OP

Query SNP	CHR	Proxy SNP	LD r^2	Major/ Minor	Allele		Dominant		Recessive	
					OR (95% CI)	p	OR (95% CI)	p	OR (95% CI)	p
rs2234693	6	rs3853250	0.98	T/G	1.01 (0.95-1.07)	0.75	1.04 (0.96-1.13)	0.36	0.96 (0.86-1.08)	0.54
rs3102735	8	rs1385504	1.00	T/A	1.09 (1.00-1.18)	0.05	1.10 (1.00-1.21)	0.04*	1.08 (0.81-1.43)	0.62
rs2073618	8	rs7464496	0.99	T/C	0.98 (0.92-1.05)	0.63	0.99 (0.91-1.07)	0.75	0.95 (0.80-1.13)	0.56
rs3736228	11	rs3781586	1.00	C/A	1.06 (0.98-1.14)	0.13	1.04 (0.95-1.13)	0.40	1.31 (1.05-1.62)	0.02*
rs35767	12	rs1101820	0.91	C/T	1.03 (0.96-1.09)	0.39	1.05 (0.97-1.14)	0.25	1.00 (0.88-1.14)	0.99
rs1800470	19	rs2241715	0.96	A/C	0.98 (0.92-1.04)	0.44	0.97 (0.89-1.07)	0.56	0.96 (0.87-1.07)	0.48

*$p<0.05$, adjusted with age, gender, and BMI.

Allele, Dominant, and Recessive genetic models were evaluated. Allele: per-minor-allele effect (0/1/2); Dominant: (Aa + aa) vs AA; Recessive: aa vs (AA + Aa).

Abbreviations: DGS, decisive gene strategy; SNPs, single-nucleotide polymorphisms; LD, linkage disequilibrium; TWB, Taiwan Biobank; CHR, chromosome

Supplementary Table 3. Logistic regression analysis of 11 identical SNPs in TWB to justify their relationship to OP stratified by male and age

SNPs	Age group	Allele		Dominant		Recessive	
		OR (95% CI)	p-value	OR (95% CI)	p-value	OR (95% CI)	p-value
rs9340799	Overall	0.94(0.85-1.03)	0.19	0.93(0.83-1.04)	0.22	0.90(0.68-1.18)	0.44
	< 65y/o	0.99(0.85-1.03)	0.84	0.99(0.88-1.13)	0.92	0.95(0.71-1.29)	0.75
	≥65 y/o	0.78(0.63-0.96)	0.02*	0.76(0.60-0.96)	0.02*	0.69(0.35-1.35)	0.28
rs1800795	Overall	0.60(0.13-2.82)	0.51	0.60(0.13-2.82)	0.51	NA	NA
	< 65y/o	0.79(0.13-2.82)	0.77	0.79(0.16-3.90)	0.77	NA	NA
	≥65 y/o	NA	NA	NA	NA	NA	NA
rs2073617	Overall	1.04(0.96-1.13)	0.32	1.01(0.91-1.13)	0.81	1.13(0.97-1.32)	0.11
	< 65y/o	1.06(0.96-1.13)	0.23	1.04(0.92-1.18)	0.54	1.14(0.96-1.35)	0.14
	≥65 y/o	0.99(0.84-1.16)	0.89	0.93(0.74-1.17)	0.52	1.10(0.81-1.51)	0.54
rs3134069	Overall	1.07(0.96-1.20)	0.20	1.06(0.94-1.19)	0.34	1.32(0.92-1.90)	0.13
	< 65y/o	1.09(0.96-1.20)	0.16	1.09(0.95-1.26)	0.21	1.24(0.80-1.90)	0.33
	≥65 y/o	1.01(0.81-1.26)	0.95	0.95(0.74-1.23)	0.72	1.56(0.78-3.11)	0.21
rs731236	Overall	1.06(0.88-1.26)	0.56	1.05(0.87-1.26)	0.64	1.64(0.56-4.81)	0.36
	< 65y/o	1.12(0.88-1.26)	0.29	1.11(0.90-1.37)	0.33	1.61(0.43-6.04)	0.48
	≥65 y/o	0.88(0.61-1.28)	0.51	0.86(0.58-1.26)	0.44	1.56(0.24-10.01)	0.64
rs7975232	Overall	1.02(0.94-1.11)	0.640	1.02(0.91-1.13)	0.77	1.06(0.87-1.29)	0.56
	< 65y/o	1.03(0.94-1.11)	0.53	1.05(0.93-1.19)	0.46	1.01(0.81-1.27)	0.92
	≥65 y/o	0.99(0.83-1.18)	0.91	0.92(0.74-1.16)	0.49	1.22(0.82-1.81)	0.32
rs1544410	Overall	1.15(0.98-1.36)	0.09	1.12(0.93-1.31)	0.20	3.02(1.28-7.10)	0.01*
	< 65y/o	1.15(0.98-1.36)	0.09	1.19(0.98-1.46)	0.09	2.53(0.93-6.83)	0.07
	≥65 y/o	0.99(0.70-1.39)	0.93	0.92(0.64-1.33)	0.66	5.37(0.99-29.15)	0.05
rs2288377	Overall	1.03(0.95-1.12)	0.46	1.04(0.93-1.16)	0.46	1.04(0.87-1.24)	0.69
	< 65y/o	1.01(0.95-1.12)	0.78	0.99(0.88-1.12)	0.92	1.08(0.89-1.33)	0.44
	≥65 y/o	1.09(0.92-1.28)	0.33	1.20(0.96-1.50)	0.10	0.89(0.61-1.30)	0.55
rs5742612	Overall	1.03(0.95-1.12)	0.46	1.04(0.93-1.16)	0.47	1.04(0.87-1.24)	0.68
	< 65y/o	1.01(0.95-1.12)	0.78	0.99(0.88-1.12)	0.92	1.08(0.89-1.33)	0.43
	≥65 y/o	1.09(0.92-1.28)	0.33	1.20(0.96-1.50)	0.10	0.89(0.61-1.30)	0.56
rs4986938	Overall	1.05(0.92-1.19)	0.49	1.07(0.93-1.23)	0.37	0.81(0.43-1.53)	0.51
	< 65y/o	0.97(0.92-1.19)	0.71	0.99(0.84-1.16)	0.88	0.68(0.32-1.45)	0.32
	≥65 y/o	1.34(1.02-1.75)	0.03*	1.38(1.03-1.84)	0.03*	1.30(0.39-4.40)	0.67
rs1256049	Overall	0.96(0.88-1.04)	0.27	0.94(0.84-1.05)	0.27	0.95(0.82-1.11)	0.55
	< 65y/o	0.99(0.88-1.04)	0.90	0.98(0.87-1.11)	0.78	1.01(0.85-1.21)	0.89
	≥65 y/o	0.85(0.72-1.00)	0.05*	0.82(0.65-1.02)	0.08	0.79(0.57-1.09)	0.15

*p<0.05, adjusted with age, gender, and BMI.

Allele, Dominant, and Recessive genetic models were evaluated. Allele: per-minor-allele effect (0/1/2); Dominant: (Aa + aa) vs AA; Recessive: aa vs (AA + Aa).

Abbreviations: DGS, decisive gene strategy; SNPs, single-nucleotide polymorphisms; TWB, Taiwan Biobank; CHR, chromosome

Supplementary Table 4. Logistic regression analysis of 11 identical SNPs in TWB to justify their relationship to OP stratified by female and age

SNPs	Age group	Allele		Dominant		Recessive	
		OR (95% CI)	p-value	OR (95% CI)	p-value	OR (95% CI)	p-value
rs9340799	Overall	0.95(0.88-1.02)	0.16	0.93(0.85-1.02)	0.12	0.97(0.79-1.20)	0.80
	< 65y/o	1.01(0.92-1.11)	0.88	0.99(0.89-1.11)	0.91	1.09(0.85-1.42)	0.49
	≥65 y/o	0.84(0.73-0.96)	<0.01*	0.81(0.69-0.95)	0.01*	0.77(0.52-1.12)	0.17
rs1800795	Overall	0.88(0.32-2.41)	0.80	0.88(0.32-2.41)	0.80	NA	NA
	< 65y/o	0.93(0.30-2.88)	0.90	0.93(0.30-2.88)	0.90	NA	NA
	≥65 y/o	0.72(0.07-7.28)	0.78	0.72(0.07-7.28)	0.78	NA	NA
rs2073617	Overall	1.01(0.95-1.08)	0.68	1.03(0.94-1.13)	0.48	0.99(0.86-1.13)	0.84
	< 65y/o	1.05(0.97-1.14)	0.25	1.07(0.95-1.20)	0.25	1.05(0.89-1.24)	0.54
	≥65 y/o	0.95(0.85-1.06)	0.37	0.97(0.83-1.13)	0.69	0.86(0.68-1.09)	0.22
rs3134069	Overall	1.08(0.98-1.18)	0.12	1.08(0.97-1.20)	0.15	1.19(0.85-1.66)	0.32
	< 65y/o	1.11(0.99-1.24)	0.08	1.13(0.99-1.28)	0.07	1.09(0.72-1.65)	0.67
	≥65 y/o	1.02(0.87-1.20)	0.84	0.99(0.83-1.18)	0.92	1.41(0.79-2.52)	0.25
rs731236	Overall	0.95(0.82-1.11)	0.50	0.96(0.82-1.13)	0.63	0.48(0.16-1.44)	0.19
	< 65y/o	1.00(0.83-1.20)	0.97	1.02(0.84-1.23)	0.86	0.47(0.13-1.70)	0.25
	≥65 y/o	0.86(0.65-1.12)	0.26	0.86(0.65-1.13)	0.28	0.52(0.06-4.69)	0.56
rs7975232	Overall	0.96(0.90-1.03)	0.30	0.94(0.86-1.03)	0.15	1.02(0.86-1.20)	0.84
	< 65y/o	0.99(0.91-1.08)	0.85	0.98(0.88-1.10)	0.77	1.01(0.82-1.24)	0.94
	≥65 y/o	0.91(0.81-1.03)	0.13	0.85(0.73-0.99)	0.04*	1.03(0.78-1.36)	0.84
rs1544410	Overall	0.96(0.83-1.11)	0.56	0.95(0.81-1.10)	0.48	1.36(0.54-3.41)	0.51
	< 65y/o	1.02(0.85-1.22)	0.85	1.02(0.85-1.23)	0.81	0.82(0.23-2.96)	0.76
	≥65 y/o	0.85(0.65-1.10)	0.21	0.81(0.62-1.06)	0.12	3.10(0.75-12.91)	0.12
rs2288377	Overall	1.04(0.97-1.12)	0.24	1.05(0.96-1.15)	0.25	1.05(0.91-1.22)	0.50
	< 65y/o	0.97(0.89-1.05)	0.43	0.97(0.87-1.08)	0.57	0.92(0.76-1.12)	0.43
	≥65 y/o	1.18(1.06-1.33)	<0.01*	1.23(1.06-1.44)	<0.01*	1.27(1.00-1.61)	0.05*
rs5742612	Overall	1.04(0.97-1.12)	0.23	1.06(0.96-1.15)	0.24	1.05(0.91-1.22)	0.51
	< 65y/o	0.97(0.89-1.05)	0.44	0.97(0.87-1.08)	0.59	0.92(0.76-1.12)	0.42
	≥65 y/o	1.18(1.06-1.33)	<0.01*	1.23(1.06-1.44)	<0.01*	1.27(1.00-1.61)	0.05*
rs4986938	Overall	1.03(0.92-1.15)	0.58	1.04(0.92-1.17)	0.56	1.02(0.66-1.57)	0.94
	< 65y/o	1.10(0.96-1.25)	0.18	1.12(0.97-1.29)	0.13	0.97(0.57-1.65)	0.92
	≥65 y/o	0.92(0.76-1.11)	0.37	0.89(0.72-1.10)	0.29	1.12(0.53-2.36)	0.77
rs1256049	Overall	0.94(0.88-1.00)	0.06	0.96(0.87-1.05)	0.34	0.85(0.74-0.97)	0.02*
	< 65y/o	0.91(0.84-0.99)	0.02*	0.90(0.81-1.01)	0.08	0.84(0.71-0.99)	0.04*
	≥65 y/o	0.99(0.89-1.11)	0.90	1.06(0.91-1.24)	0.46	0.86(0.69-1.08)	0.20

*p<0.05, adjusted with age, gender, and BMI.

Allele, Dominant, and Recessive genetic models were evaluated. Allele: per-minor-allele effect (0/1/2); Dominant: (Aa + aa) vs AA; Recessive: aa vs (AA + Aa).

Abbreviations: DGS, decisive gene strategy; SNPs, single-nucleotide polymorphisms; TWB, Taiwan Biobank; CHR, chromosome

Supplementary Table 5. Logistic regression analysis of 6 high LD SNPs to justify their relationship to OP stratified by male and age

Query SNP	CHR	Proxy SNP	LD r^2	Major/ Minor	Age group	Allele		Dominant		Recessive	
						OR (95% CI)	p	OR (95% CI)	p	OR (95% CI)	p
rs2234693	6	rs3853250	0.98	T/G	Overall	1.00 (0.92-1.08)	0.95	1.02 (0.91-1.14)	0.80	0.96 (0.83-1.12)	0.64
					< 65y/o	1.02 (0.92-1.08)	0.69	1.06 (0.94-1.21)	0.35	0.96 (0.81-1.14)	0.63
					≥65 y/o	0.94 (0.79-1.10)	0.43	0.88 (0.70-1.11)	0.28	1.00 (0.72-1.38)	0.99
rs3102735	8	rs1385504	1.00	T/A	Overall	1.06 (0.96-1.18)	0.26	1.06 (0.94-1.19)	0.37	1.23 (0.86-1.74)	0.25
					< 65y/o	1.08 (0.96-1.18)	0.21	1.09 (0.95-1.25)	0.24	1.17 (0.78-1.77)	0.45
					≥65 y/o	1.00 (0.80-1.24)	1.00	0.96 (0.75-1.23)	0.75	1.37 (0.70-2.71)	0.36
rs2073618	8	rs7464496	0.99	T/C	Overall	1.00 (0.91-1.09)	0.91	0.99 (0.89-1.11)	0.88	1.01 (0.80-1.26)	0.96
					< 65y/o	1.00 (0.91-1.09)	0.99	1.00 (0.89-1.14)	0.97	0.99 (0.76-1.28)	0.93
					≥65 y/o	0.98 (0.82-1.18)	0.84	0.96 (0.77-1.20)	0.71	1.07 (0.67-1.70)	0.78
rs3736228	11	rs3781586	1.00	C/A	Overall	1.11 (1.00-1.22)	0.04*	1.09 (0.97-1.21)	0.13	1.40 (1.05-1.85)	0.02*
					< 65y/o	1.08 (1.00-1.22)	0.17	1.05 (0.93-1.20)	0.43	1.41 (1.03-1.93)	0.03*
					≥65 y/o	1.20 (0.98-1.47)	0.08	1.22 (0.97-1.54)	0.09	1.35 (0.72-2.53)	0.35
rs35767	12	rs1101820	0.91	C/T	Overall	1.07 (0.99-1.16)	0.10	1.13 (1.01-1.26)	0.03*	1.01 (0.86-1.19)	0.90
					< 65y/o	1.07 (0.99-1.16)	0.15	1.12 (0.99-1.27)	0.08	1.03 (0.85-1.24)	0.76
					≥65 y/o	1.06 (0.90-1.25)	0.46	1.15 (0.92-1.45)	0.22	0.95 (0.67-1.33)	0.74
rs1800470	19	rs2241715	0.96	A/C	Overall	0.99 (0.92-1.07)	0.82	1.02 (0.92-1.15)	0.76	0.95 (0.83-1.09)	0.45
					< 65y/o	1.04 (0.92-1.07)	0.44	1.07 (0.93-1.22)	0.34	1.02 (0.88-1.19)	0.80
					≥65 y/o	0.86 (0.73-1.01)	0.06	0.87 (0.69-1.11)	0.26	0.73 (0.54-0.99)	0.04*

*p<0.05, adjusted with age, gender, and BMI.

Allele, Dominant, and Recessive genetic models were evaluated. Allele: per-minor-allele effect (0/1/2); Dominant: (Aa + aa) vs AA; Recessive: aa vs (AA + Aa).

Abbreviations: DGS, decisive gene strategy; SNPs, single-nucleotide polymorphisms; LD, linkage disequilibrium; TWB, Taiwan Biobank; CHR, chromosome

Supplementary Table 6. Logistic regression analysis of 6 high LD SNPs to justify their relationship to OP stratified by female and age

Query SNP	CHR	Proxy SNP	LD r^2	Major/ Minor	Age group	Allele		Dominant		Recessive	
						OR (95% CI)	p	OR (95% CI)	p	OR (95% CI)	p
rs2234693	6	rs3853250	0.98	T/G	Overall	0.98 (0.91-1.04)	0.46	0.97 (0.88-1.06)	0.46	0.97 (0.86-1.10)	0.66
					< 65y/o	1.01 (0.93-1.10)	0.81	1.03 (0.92-1.16)	0.57	0.98 (0.84-1.14)	0.77
					≥65 y/o	0.92 (0.82-1.02)	0.12	0.86 (0.73-1.00)	0.05	0.97 (0.78-1.20)	0.75
rs3102735	8	rs1385504	1.00	T/A	Overall	1.08 (0.99-1.19)	0.09	1.09 (0.98-1.21)	0.10	1.15 (0.84-1.59)	0.37
					< 65y/o	1.10 (0.98-1.23)	0.09	1.12 (0.99-1.27)	0.07	1.06 (0.72-1.57)	0.76
					≥65 y/o	1.05 (0.90-1.22)	0.56	1.03 (0.86-1.22)	0.76	1.36 (0.79-2.35)	0.27
rs2073618	8	rs7464496	0.99	T/C	Overall	0.98 (0.91-1.05)	0.56	0.98 (0.90-1.07)	0.66	0.94 (0.77-1.15)	0.55
					< 65y/o	1.00 (0.91-1.09)	0.95	1.01 (0.90-1.13)	0.91	0.95 (0.75-1.21)	0.69
					≥65 y/o	0.94 (0.83-1.07)	0.35	0.93 (0.80-1.09)	0.36	0.92 (0.65-1.31)	0.65
rs3736228	11	rs3781586	1.00	C/A	Overall	1.05 (0.97-1.14)	0.22	1.05 (0.96-1.16)	0.28	1.12 (0.88-1.43)	0.36
					< 65y/o	1.08 (0.98-1.20)	0.12	1.07 (0.95-1.20)	0.28	1.31 (0.98-1.75)	0.07
					≥65 y/o	1.00 (0.87-1.15)	0.99	1.03 (0.88-1.21)	0.73	0.80 (0.51-1.26)	0.34
rs35767	12	rs1101820	0.91	C/T	Overall	1.02 (0.96-1.09)	0.54	1.04 (0.95-1.14)	0.41	1.00 (0.87-1.15)	0.98
					< 65y/o	0.96 (0.88-1.04)	0.30	0.96 (0.86-1.07)	0.47	0.91 (0.77-1.09)	0.30
					≥65 y/o	1.14 (1.02-1.28)	0.02*	1.21 (1.03-1.41)	0.02*	1.16 (0.92-1.44)	0.20
rs1800470	19	rs2241715	0.96	A/C	Overall	0.98 (0.92-1.05)	0.56	0.98 (0.89-1.08)	0.70	0.97 (0.86-1.08)	0.56
					< 65y/o	0.93 (0.86-1.01)	0.08	0.90 (0.80-1.02)	0.09	0.92 (0.80-1.06)	0.26
					≥65 y/o	1.08 (0.97-1.20)	0.17	1.15 (0.97-1.36)	0.10	1.05 (0.87-1.27)	0.61

*p<0.05, adjusted with age, gender, and BMI.
Allele, Dominant, and Recessive genetic models were evaluated. Allele: per-minor-allele effect (0/1/2); Dominant: (Aa + aa) vs AA; Recessive: aa vs (AA + Aa).
Abbreviations: DGS, decisive gene strategy; SNPs, single-nucleotide polymorphisms; LD, linkage disequilibrium; TWB, Taiwan Biobank; CHR, chromosome

Supplementary Table 7. Allele frequencies in TWB and 1000 Genomes EAS and EUR for 11 identical and 6 high LD SNPs

SNPs	Aligned result	TWB (n=262,096) [a]		1000 Genomes EAS (n=1,008) [b]		1000 Genomes EUR (n=1,006) [b]	
		Ref	Alt	Ref	Alt	Ref	Alt
rs9340799_A/G	Identical	A=0.7887	G=0.2113	A=0.8065	G=0.1935	A=0.6918	G=0.3082
rs1800795_C/G	Identical	C=0.0010	G=0.9990	C=0.0010	G=0.9990	C=0.4155	G=0.5845
rs2073617_G/A	Identical	G=0.3694	A=0.6306	G=0.3542	A=0.6458	G=0.4891	A=0.5109
rs3134069_A/C	Identical	A=0.8673	C=0.1327	A=0.8780	C=0.1220	A=0.9563	C=0.0437
rs731236_A/G	Identical	A=0.9507	G=0.0493	A=0.9325	G=0.0675	A=0.6004	G=0.3996
rs7975232_C/A	Identical	C=0.7083	A=0.2917	C=0.7093	A=0.2907	C=0.4453	A=0.5547
rs1544410_C/T	Identical	C=0.9477	T=0.0523	C=0.9355	T=0.0645	C=0.5964	T=0.4036
rs2288377_A/T	Identical	A=0.6899	T=0.3101	A=0.7063	T=0.2937	A=0.9841	T=0.0159
rs5742612_A/G	Identical	A=0.6899	G=0.3101	A=0.7063	G=0.2937	A=0.9563	G=0.0437
rs4986938_C/T	Identical	C=0.9086	T=0.0914	C=0.8681	T=0.1319	C=0.6233	T=0.3767
rs1256049_C/T	Identical	C=0.6295	T=0.3705	C=0.5972	T=0.4028	C=0.9642	T=0.0358
rs3853250_T/G	High LD	T=0.6119	G=0.3881	T=0.6012	G=0.3988	T=0.5765	G=0.4235
rs1385504_T/A	High LD	T=0.8596	A=0.1404	T=0.8710	A=0.1290	T=0.8678	A=0.1322
rs7464496_T/C	High LD	T=0.2439	C=0.7561	T=0.2381	C=0.7619	T=0.5358	C=0.4642
rs3781586_C/A	High LD	C=0.8101	A=0.1899	C=0.7966	A=0.2034	C=0.8459	A=0.1541
rs1101820_C/T	High LD	C=0.3497	T=0.6503	C=0.3522	T=0.6478	C=0.1690	T=0.8310
rs2241715_A/C	High LD	A=0.5565	C=0.4435	A=0.5476	C=0.4524	A=0.3141	C=0.6859

SNPs are listed in order of chromosome position.

[a] TWB allele frequencies were calculated from the TWB genotype reference dataset (n = 262,096), whereas association analyses were performed in the analytic sample (n = 26,225).

[b] 1000 Genomes EAS and EUR allele frequencies were obtained via NCBI dbSNP (1000 Genomes Project population data). EUR represents individuals of European ancestry (often termed 'Caucasian' in prior literature). Ref_allele and Alt_allele were reported and aligned across datasets based on dbSNP annotations.

Abbreviations: TWB, Taiwan Biobank; EAS, East Asians; EUR, Europeans; LD, linkage disequilibrium; SNPs, single-nucleotide polymorphisms; Ref, reference allele; Alt, alternative allele.

Supplementary Table 8. Summary statistics for TWB validation of DGS-prioritized SNPs (allelic model)

SNPs from DGS	rs number	TWB alignment	Validation result	Overall				Male				Female			
				**OR (95% CI)**	**ln(OR)**	**SE (ln[OR])**	**p**	**OR (95% CI)**	**ln(OR)**	**SE (ln[OR])**	**p**	**OR (95% CI)**	**ln(OR)**	**SE (ln[OR])**	**p**
ESR1 XbaI	rs9340799	Identical	Consistent	1.01 (0.94-1.09)	0.01	0.0378	0.72	0.94 (0.85-1.03)	-0.0619	0.049	0.19	0.95 (0.88-1.02)	-0.0513	0.0377	0.16
OPG T950C	rs2073617	Identical	Consistent	1.03 (0.97-1.10)	0.0296	0.0321	0.33	1.04 (0.96-1.13)	0.0392	0.0416	0.32	1.01 (0.95-1.08)	0.01	0.0327	0.68
VDR TaqI	rs731236	Identical	Consistent	1.06 (0.93-1.22)	0.0583	0.0692	0.40	1.06 (0.88-1.26)	0.0583	0.0916	0.56	0.95 (0.82-1.11)	-0.0513	0.0772	0.50
VDR ApaI	rs7975232	Identical	Consistent	1.01 (0.94-1.07)	0.01	0.033	0.85	1.02 (0.94-1.11)	0.0198	0.0424	0.64	0.96 (0.90-1.03)	-0.0408	0.0344	0.30
VDR BsmI	rs1544410	Identical	Consistent	1.10 (0.97-1.26)	0.0953	0.0667	0.14	1.15 (0.98-1.36)	0.1398	0.0836	0.09	0.96 (0.83-1.11)	-0.0408	0.0742	0.56
IL6 G174C	rs1800795	Identical	Consistent	0.72 (0.32-1.64)	-0.3285	0.4169	0.44	0.60 (0.13-2.82)	-0.5108	0.7849	0.51	0.88 (0.32-2.41)	-0.1278	0.5151	0.80
IGF1	rs5742612	Identical	Consistent	1.02 (0.96-1.09)	0.0198	0.0324	0.58	1.03 (0.95-1.12)	0.0296	0.042	0.46	1.04 (0.97-1.12)	0.0392	0.0367	0.23
ESR1 PvuII	rs2234693	High LD, rs3853250 (r^2=0.98)	Consistent	1.01 (0.95-1.07)	0.01	0.0303	0.75	1.00 (0.92-1.08)	0.0	0.0409	0.95	0.98 (0.91-1.04)	-0.0202	0.0341	0.46
OPG G1181C	rs2073618	High LD, rs7464496 (r^2=0.99)	Consistent	0.98 (0.92-1.05)	-0.0202	0.0337	0.63	1.00 (0.91-1.09)	0.0	0.046	0.91	0.98 (0.91-1.05)	-0.0202	0.0365	0.56
LRP5 A1330V	rs3736228	High LD, rs3781586 (r^2=1.00)	Consistent	1.06 (0.98-1.14)	0.0583	0.0386	0.13	1.11 (1.00-1.22)	0.1044	0.0507	0.04*	1.05 (0.97-1.14)	0.0488	0.0412	0.22
ESR2 RsaI	rs1256049	Identical	Inconsistent	0.95 (0.90-1.01)	-0.0513	0.0294	0.12	0.96 (0.88-1.04)	-0.0408	0.0426	0.27	0.94 (0.88-1.00)	-0.0619	0.0326	0.06
ESR2 AluI	rs4986938	Identical	Inconsistent	1.02 (0.92-1.13)	0.0198	0.0524	0.71	1.05 (0.92-1.19)	0.0488	0.0656	0.49	1.03 (0.92-1.15)	0.0296	0.0569	0.58
IGF1	rs2288377	Identical	Inconsistent	1.02 (0.96-1.09)	0.0198	0.0324	0.58	1.03 (0.95-1.12)	0.0296	0.042	0.46	1.04 (0.97-1.12)	0.0392	0.0367	0.24
OPG A163G	rs3102735	High LD, rs1385504 (r^2=1.00)	Inconsistent	1.09 (1.00-1.18)	0.0862	0.0422	0.05	1.06 (0.96-1.18)	0.0583	0.0526	0.26	1.08 (0.99-1.19)	0.077	0.0469	0.09
IGF1	rs35767	High LD, rs1101820 (r^2=0.91)	Inconsistent	1.03 (0.96-1.09)	0.0296	0.0324	0.39	1.07 (0.99-1.16)	0.0677	0.0404	0.10	1.02 (0.96-1.09)	0.0198	0.0324	0.54
TGFβ1 T869C	rs1800470	High LD, rs2241715 (r^2=0.96)	Inconsistent	0.98 (0.92-1.04)	-0.0202	0.0313	0.44	0.99 (0.92-1.07)	-0.0101	0.0385	0.82	0.98 (0.92-1.05)	-0.0202	0.0337	0.56
OPG T245G	rs3134069	Identical	Novel	1.09 (1.00-1.19)	0.0862	0.0444	<0.05*	1.07 (0.96-1.20)	0.0677	0.0569	0.20	1.08 (0.98-1.18)	0.077	0.0474	0.12

Effect sizes are odds ratios (ORs) from case–control logistic regression (per-minor-allele effect) in the overall sample and sex-stratified analyses (male and female). Standard errors (SE) are reported for ln(OR). When SE was not directly exported, it was derived from the 95% CI as: SE = (ln[Upper] − ln[Lower])/(2 × 1.96).

*$p < 0.05$. Abbreviations: CI, confidence interval; DGS, decisive gene strategy; LD, linkage disequilibrium; OR, odds ratio; SE, standard error; SNP, single-nucleotide polymorphism; TWB, Taiwan Biobank.
